# *CTNNB1* p.D32A (c.95A > C) somatic mutation in stage I grade 1 endometrioid endometrial carcinoma with lung metastasis: a case report

**DOI:** 10.1186/s12920-023-01570-3

**Published:** 2023-06-16

**Authors:** Lan Zhong, Wei Jiang, RutieYin, Hui Liu, Liang Song

**Affiliations:** 1 Department of Obstetrics and Gynecology, West China Second University Hospital of Sichuan University, No. 20 The Third Section of South Renmin Road, Chengdu, 610041 Sichuan China; 2 Key Laboratory of Birth Defects and Related Diseases of Women and Children (Sichuan University), Ministry of Education, Chengdu, China; 3 Department of Pathology, West China Second University Hospital, Sichuan University, Chengdu, China

**Keywords:** Endometrial cancer, Next-generation sequencing, Lung Metastasis, Case report

## Abstract

**Background:**

Most endometrial cancers are of low histological grade and uterine-confined, with a high 5-year survival rate. However, a small subset of women with low-grade and early-stage endometrioid endometrial cancer experience recurrence and death; thus, a more precise risk-stratification is needed.

**Case presentation:**

A 29-year-old woman presented with abnormal vaginal bleeding and was diagnosed with FIGO grade 1 endometrioid endometrial carcinoma by curettage. Comprehensive cancer staging including pelvic and para-aortic lymphadenectomy was then performed. Postoperative pathological findings suggested an FIGO grade 1 endometrioid endometrial carcinoma infiltrating the superficial muscle layer. The patient did not receive adjuvant therapy. After 4 years of follow-up, the patient returned to our institution with lung metastasis. She underwent thoracoscopic resection of the affected lobes, followed by six cycles of combined chemotherapy of paclitaxel and carboplatin. Next-generation sequencing showed that the primary and lung metastatic tumors shared 4 mutations: *PTEN* (p.P248Lfs*8), *CTNNB1* (p.D32A), *BCOR* (p.N1425S) and *CBL* (p.S439N). Immunohistochemistry revealed nuclear location of β–catenin in the primary and lung metastatic tumor samples, indicating abnormal activation of β–catenin.

**Conclusion:**

*CTNNB1*p.D32A (c.95A > C) mutation may be related to lung metastasis in this patient with low-grade early-stage endometrioid endometrial carcinoma.

**Supplementary Information:**

The online version contains supplementary material available at 10.1186/s12920-023-01570-3.

## Core tip

A small subset of women with low-grade and early-stage endometrioid endometrial cancer experience recurrence or death. The copy number (CN)-low (endometrioid) group demonstrated a high frequency of *CTNNB1* mutations (52%), while studies on the relationship between *CTNNB1* mutation and prognosis of the CN-low (endometrioid) patients have different conclusions.. It may be necessary to subtype the *CTNNB1*-mutation group correlated with the poor outcome of endometrioid adenocarcinoma. Our patient harbored *CTNNB1*p.D32A (c.95A > C) somatic mutation and experienced lung metastasis. Moreover, we observed nuclear location of β-catenin in the primary and metastatic tumor samples. We hypothesize that D32A is involved in metastasis through activating β-catenin.

## Background

Endometrial cancer is the most common malignancy of the female genital tract. Most patients are diagnosed with low-grade, uterine-confined disease and have a 5-year survival rate of over 90% [1]. However, despite this generally good prognosis, a small subset of women with low-grade and low-stage endometrial cancer experience disease recurrence and death. The recurrence rate of early-stage disease ranges from 2 to 26% in the reported literature, and varies widely across different histologic subtypes [2]. A recent retrospective study reported that 2.9% of patients with FIGO grade 1, non-myoinvasive tumors, and absence of lymphovascular space invasion developed recurrence [3]. In endometrioid carcinoma, risk-stratification is especially difficult and critical for early-stage, grade 1 tumors [4, 5]. In 2013, a large scale analysis by the Cancer Genome Atlas (TCGA) project, proposed a new genomic classification for endometrial cancer: POLE-mutated (ultramutated), microsatellite instability-high (MSI-high, hypermutated), copy-number (CN) low (endometrioid-like), and CN high (serous-like). The CN-low (endometrioid) group demonstrated a high frequency of *CTNNB1* mutations (52%) and *PTEN* mutations. However, the heterogeneous clinical course of endometrioid carcinoma is still an obstacle to individualized treatment.Mutations in the *CTNNB1* exon 3 hotspot were suggested to be drivers of a more aggressive subtype of low-grade, early-stage endometrioid endometrial carcinoma [6–8]. However, a study failed to confirm this conclusion [9]. Herein, we describe the case of a 29-year-old patient with stage I grade 1 endometrioid endometrial carcinoma (CN-low), returned to our institution with lung metastasis, harbored CTNNB1p.D32A (c.95A > C) somatic mutation.

## Case presentation

### Chief complaints

A 29-year-old woman with stage I grade 1 endometrioid endometrial carcinoma presented with lung metastasis.

### History of present illness

**Fig. 1:**
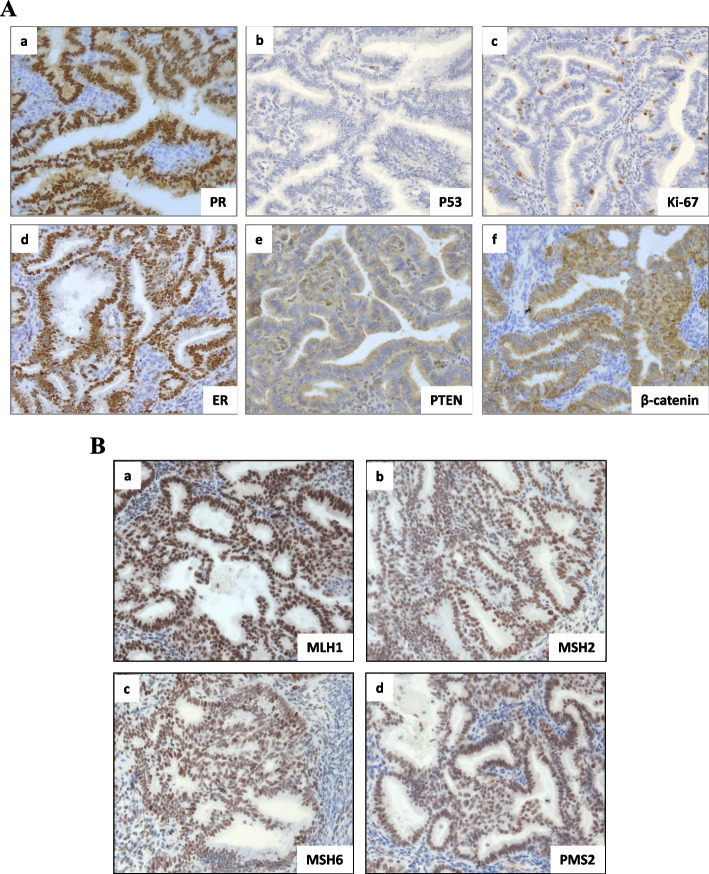
**A**: Immunohistochemical findings. **a **& **d**: the tumor tissue showed high expression of estrogen and progesterone receptors (ER +  +  + , PR +  + +). **b**: immunohistochemical staining of p53 showed weak and heterogeneous staining, indicating a wild-type pattern. **c**: Ki67 was 25% positive. **e**: partial loss of expression of PTEN (+ +). **e**: clustered nuclear staining of β-catenin (+ + +) (200 ×). **B**: Immunohistochemical staining of mismatch repair proteins (**a**. MLH1, **b**. MSH2, **c**. MSH6, **d**. PMS2), all showing retention of nuclear staining (200 ×)

**Fig. 2:**
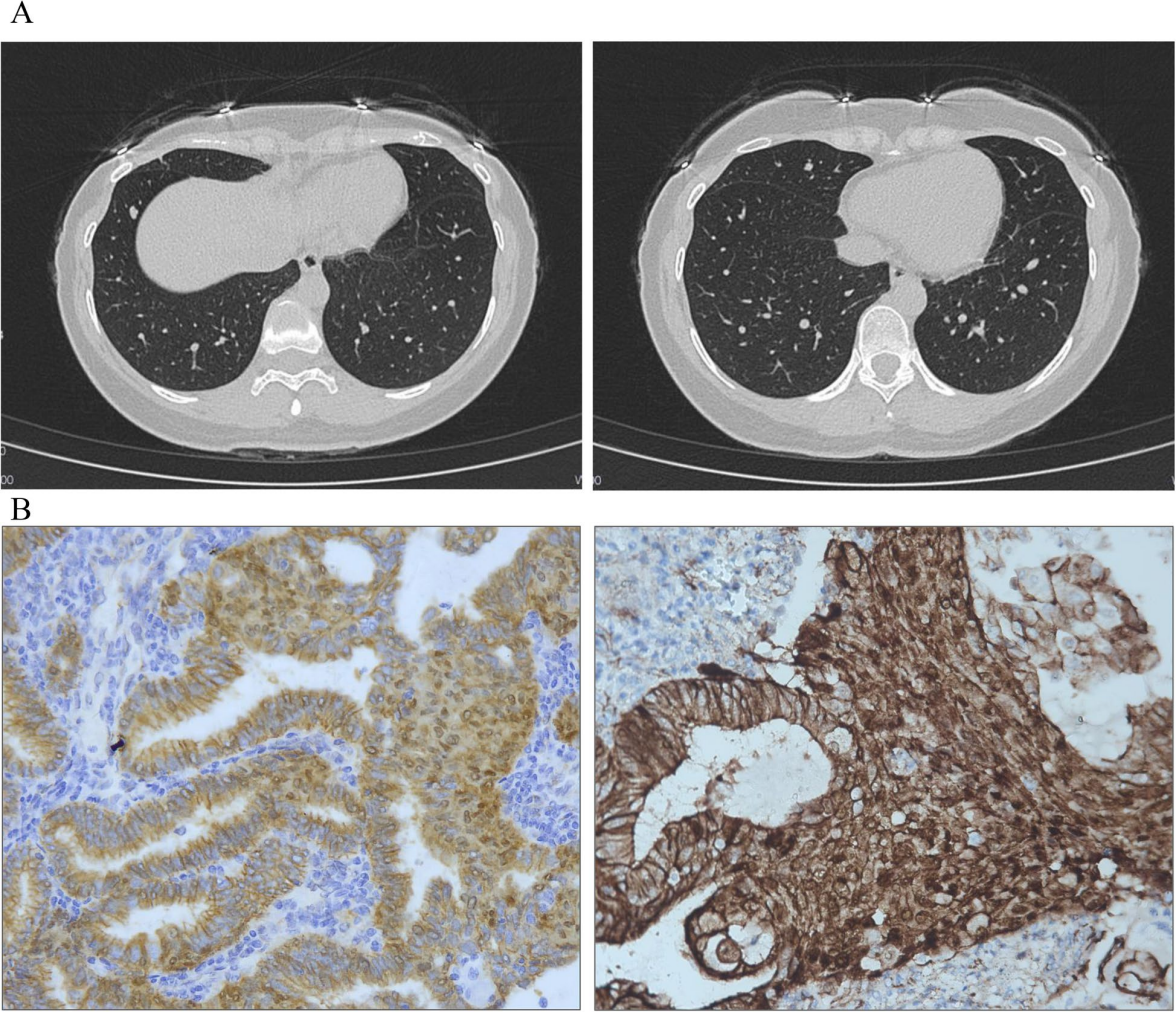
CT findings. **A**: CT scan of the chest showed lung metastasis. Left: Cross-section of a high-density pulmonary nodule in the anterior basal region of the right lower lobe. Right: Cross-section of a high-density pulmonary nodule in the right middle lobe of the lung. **B**: β-catenin was evaluated by IHC in primary and metastatic tumors. Clustered nuclear location of β-catenin in primary tumor (**a**) and lung metastatic tumor (**b**) (400x)

In 2015, the patient experienced abnormal vaginal bleeding. Her BMI was 20.3 kg/m^2^. G_2_P_1_^+1^. Ultrasound examination revealed a 3^+^cm mass in the uterine cavity. In February 2015, she underwent curettage and was diagnosed with FIGO grade 1 endometrioid carcinoma. Magnetic resonance imaging suggested the possibility of cervical invasion. Thoracic computed tomography (CT) was negative. She subsequently underwent comprehensive cancer staging including laparoscopic radical hysterectomy, bilateral salpingo-oophorectomy, pelvic and para-aortic lymphadenectomy, with pelvic washing. Postoperative pathological examinations showed the followings: FIGO grade 1 endometrioid adenocarcinoma infiltrating the superficial muscle layer, and no lymphovascular space invasion. The ovaries and fallopian tubes showed no malignancy. Left and right pelvic and para-aortic lymph nodes showed no evidence of malignancy in 26 examined nodes. Peritoneal cytology was negative. A diagnosis of FIGO (2018) stage I endometrioid adenocarcinoma was made. Immunohistochemical (IHC) staining showed that the tumor tissue had a high expression of estrogen receptors (ERs) and progesterone receptors (PRs) (Fig. 1A) and normal DNA mismatch repair (MMR) protein expression (Fig. 1B). IHC staining for p53 showed a wild-type pattern (Fig. 1A). Notably, clustered nuclear staining of β-catenin was detected (Fig. 1A). A postoperative CT scan of the chest, abdomen and pelvis was negative. According to the NCCN Guidelines at that time, the patient did not receive adjuvant therapy. The patient was followed up regularly in another hospital. In June 2019, she returned to our hospital with an irregular cough without apparent cause. A pulmonary CT scan showed multiple high-density pulmonary nodules in the anterior basal region of the right lower lobe and right middle lobe of the lung, indicating metastatic tumors (Fig. 2A). She then underwent thoracoscopic resection of the affected lobes. IHC confirmed an FIGO grade 1 endometrioid adenocarcinoma infiltrating the lung tissue. The patient was then treated with six cycles of combined chemotherapy of paclitaxel and carboplatin. Combined with the computed tomography scan result and the cancer antigen 125, complete remission following treatment was confirmed. The patient was followed up regularly for 18 months after completing chemotherapy, and no tumor recurrence occurred during this period.

### History of past illness

The patient denied a history of systematic diseases and a history of smoking.

### Personal and family history

The patient denied a personal and family history of related diseases.

### Physical examination

There was no physical examination.

### Laboratory examinations

Routine blood test, liver and kidney function, and tumor markers like cancer antigen 125 (CA125), cancer antigen 199 (CA199), carcinoembryonic antigen(CEA), alpha-fetoprotein (AFP) were normal.

### Imaging examinations

A pulmonary CT scan showed multiple high-density pulmonary nodules in the anterior basal region of the right lower lobe and right middle lobe of the lung, indicating metastatic tumors (Fig. 2A).

### Genetic alterations

**Fig. 3:**
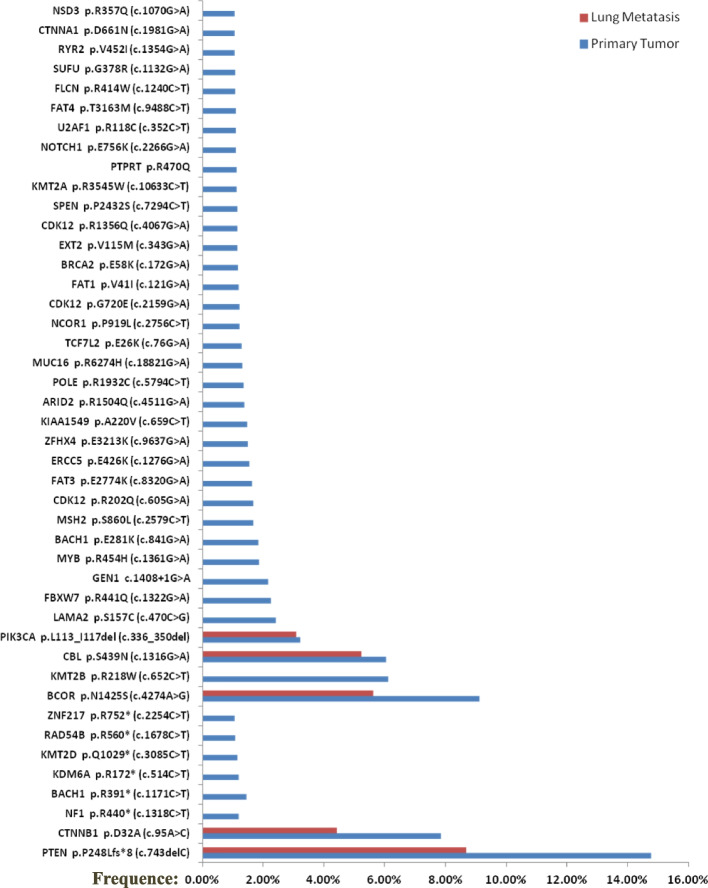
Mutational signature of the primary and lung metastatic tumors. Somatic mutations of 44 genes were detected in primary tumor samples. Lung metastasis carried mutations of *PTEN*(p.P248Lfs*8), *CTNNB1* (p.D32A), *BCOR* (p.N1425S), and *CBL* (p.S439N), which were shared by a somatic mutation

To gain further insight into the driver gene mutation mediating metastasis, targeted next-generation sequencing (NGS) of 688 cancer-related genes was performed on both primary tumor and lung metastasis specimens (Supplementary Table 1). DNA was extracted from the paraffin-embedded specimens and blood samples from the patient to evaluate somatic and germline mutations, respectively. The variant frequency for each sample was calculated as the percent variant reads from total reads. The data showed that there was no germline mutation in this patient. Somatic mutations of 44 genes were detected in primary tumor samples. Mutations of *PTEN* (p.P248Lfs*8), *CTNNB1* (p.D32A), *BCOR* (p.N1425S) and *CBL* (p.S439N) were detected in the lung metastasis, which were shared by primary tumors (Fig. 3, Supplementary Table 2). Exonic sequence data across all genes showed no MMR deficiency signature, ultramutated phenotype in *POLE* or *TP*53 mutation for both primary and metastatic tumors (Table 1). According to genomic classification, the patient belonged to the CN-low group.

**Table 1:** Molecular characteristics of primary tumor and lung metastasis

	Primary tumor	Lung metastasis
P53 mutation (PCR)	-	-
P53 mutation (IHC)	-	N.A
MSI (PCR)	MSS	MSS
MSI/MMR (IHC)	Normal	normal
POLE ultramutation (PCR)	-	-
TMB	6.45 Muts/Mb	1.79 Muts/Mb
Selected variants (NGS)	*PTEN*, *CTNNB1* etc	*PTEN*, *CTNNB1* etc

### Protein expression profile (immunohistochemistry)

IHC of the postoperative sample after primary surgery revealed the following: ER +  +  + , PR +  +  + , β-catenin +  +  + , PTEN +  + , P53-, and Ki67 25% (Fig. 1A). The expressions of four MMR proteins (MSH2, MLH1, MSH6 and PMS2) were retained in primary and metastatic tumor tissues, suggestive of microsatellite stable carcinoma, which was consistent with the NGS results (Fig. 1B). IHC of pulmonary metastatic lesion was as follws: Napsin-A (-), TTF-1 (-), PAX-8 ( +), Ck7 (-), ER (+ +), and PR (+ + +).We evaluated β-catenin by IHC in primary and metastatic tumors, and found clustered nuclear location of β-catenin in both the primary tumor and lung metastatic tumor (Fig. 2B), indicating high β-catenin activity.

### TCGA database screening

Screening of gene mutations in endometrioid carcinoma was performed using the TCGA. The results showed that there are 130 mutations of *CTNNB1* in 399 cases of endometrioid adenocarcinoma, including 2 cases with mutation of *D32A*.

## Discussion and conclusion

The incidence of *PTEN* alterations in endometrial cancer according to the COSMIC and TCGA datasets is 43% and 46.6%, respectively [10]. There is solid evidence to indicate that *PTEN* loss occurs during the neoplastic process of endometrioid endometrial cancer [11, 12]. To date, there is no definite evidence to show that *BCOR* or *CBL* is involved in tumorigenesis or progression of endometrial cancer. In this patient, *CTNNB1* mutation may be related to lung metastasis.

As previously mentioned, the CN-low (endometrioid) group demonstrated a high frequency of *CTNNB1* mutations (52%), while the recurrence rate in this group is far lower than that. Studies on the relationship between *CTNNB1* mutation and prognosis of the CN-low (endometrioid) group show different conclusions, most studies suggest that *CTNNB1* mutation correlates with worse outcomes [7, 13], but there are also opposite results where *CTNNB1* mutation is not correlated with the prognosis of endometrioid adenocarcinoma [9]. It may be necessary to subtype the *CTNNB1*-mutation group and determine the exact mutant(s) correlated with the poor outcome of endometrioid adenocarcinoma.

Exon 3 of *CTNNB1*, encodes protein β-catenin which is involved in both cadherin-mediated cell–cell adhesion and the Wnt signaling cascade, and is reported to have several mutational hotspots at or near phosphorylation sites, which could lead to β-catenin activation. Activated β-catenin was proved to drive tumorigenesis in multiple cancers [14], especially colorectal cancer [15] and endometrial cancer [16]. In addition to disruption of key serine and threonine residues, mutations are frequently reported in other residues in exon 3 that are not kinase substrates. The most frequently mutated non-serine/non-threonine residues are D32 and G34 [17]. *CTNNB1* mutant D32 lies within the ubiquitination recognition motif of β-catenin. The *CTNNB1* mutant D32A resulted in decreased levels of β-catenin ubiquitination, leading to increased β-catenin in the cytoplasm, finally inducing nuclear translocation. D32 is ranked within the top six mutations identified in human tumors [17, 18]. D32A resulted in decreased levels of β-catenin ubiquitination, and demonstrated transformation in all assays. Elayne Provost showed that stable cell lines harboring D32A-mutated β-catenin were highly transformed compared to S33A and G34. D32A mutation altered the morphology of colonies in soft agar, increased invasion and migration in a wounding assay and increased growth and cell shedding at confluence [17]. It may be concluded that D32A represents a highly transforming mutation in exon 3 hotspots.

Our patient harbored *CTNNB1* p.D32A (c.95A > C) somatic mutation and experienced lung metastasis. Moreover, we observed nuclear location of β-catenin in both primary and metastatic tumor samples. *CTNNB1* p.D32A (c.95A > C) mutation may be related to lung metastasis in this patient with low-grade early-stage endometrioid endometrial carcinoma. D32A represents a highly transforming mutation possibly through activating β-catenin. Despite all this, the CTNNB1 p.D32A (c.95A > C) variant in endometrial cancer is not found in the public database to date and that the possible phenotypic impact of this variant needs to be further validated in a larger number of cases.

## Supplementary Information

**Additional file 1:** **Supplementary Table 1.** All genes sequenced.

**Additional file 2:** **Supplementary Table 2.** list of mutations in primary and lung metastatic tumors.

## Data Availability

All data related to this case report are available from the corresponding author by request.

## Abbreviations

CN: Copy number
POLE: Polymerase-ε
MSI: Microsatellite instability
CT: Computed tomography
IHC: Immunohistochemical
ER: Estrogen receptors
PR: Progesterone receptors
MMR: DNA mismatch repair
TCGA: The Cancer Genome Atlas
NCCN: National Comprehensive Cancer Network
FIGO: International Federation of Gynecology and Obstetrics

## References

[1] Siegel Rl, Miller Kd, Jemal A (2019). Cancer Statistics, 2019. Ca Cancer J Clin.

[2] Fujimoto T, Nanjyo H, Fukuda J (2009). Endometrioid uterine cancer: histopathological risk factors of local and distant recurrence. Gynecol Oncol.

[3] Stasenko M, Feit N, Ssk L (2020). Clinical patterns and genomic profiling of recurrent ‘Ultra-Low Risk’ endometrial cancer. Int J Gynecol Cancer.

[4] Stelloo E, Nout RA, Osse EM (2016). Improved Risk Assessment By Integrating Molecular And Clinicopathological Factors In Early-Stage Endometrial Cancer-Combined Analysis Of The Portec Cohorts. Clin Cancer Res.

[5] Jt V, Ek L, Ea G (2019). Diagnosis and management of a recurrent polymerase-epsilon (Pole)-mutated endometrial cancer. Gynecol Oncol.

[6] Liu Y, Patel L, Mills GB, Lu KH, Sood AK, Ding L, Kucherlapati R, Mardis ER, Levine DA, Shmulevich I, Broaddus RR, Zhang W. Clinical significance of CTNNB1 mutation and Wnt pathway activation in endometrioid endometrial carcinoma. J Natl Cancer Inst. 2014;106(9):dju245. 10.1093/jnci/dju245. 10.1093/jnci/dju245 PMC4200060 25214561

[7] Kc K, Gn K, Bm F (2017). Ctnnb1 (Beta-Catenin) mutation identifies low grade, early stage endometrial cancer patients at increased risk of recurrence. Mod Pathol.

[8] Mr M, Davies Kd, Ac W (2019). Molecular markers in recurrent stage I, Grade 1 endometrioid endometrial cancers. Gynecol Oncol.

[9] Costigan DC, Dong F, Nucci MR, Howitt BE (2020). Clinicopathologic And Immunohistochemical Correlates Of Ctnnb1 Mutated Endometrial Endometrioid Carcinoma. Int J Gynecol Pathol.

[10] Nero C, Ciccarone F, Pietragalla A, Scambia G. PTEN and Gynecological Cancers. Cancers (Basel). 2019;11(10):1458. 10.3390/cancers11101458. 10.3390/cancers11101458 PMC6826459 31569439

[11] Raffone A, Travaglino A, Saccone G (2019). Pten expression in endometrial hyperplasia and risk of cancer: a systematic review and meta-analysis. Arch Gynecol Obstet.

[12] Cheung LW, Hennessy BT, Li J (2011). High Frequency Of Pik3r1 And Pik3r2 Mutations In Endometrial Cancer Elucidates A Novel Mechanism For Regulation Of Pten Protein Stability. Cancer Discov.

[13] Myers  A, Barry WT, Hirsch MS, Matulonis U, Lee L (2014). Β-Catenin Mutations In Recurrent Figo Ia Grade I Endometrioid Endometrial Cancers. Gynecol Oncol.

[14] Gao C, Wang Y, Broaddus R, Sun L, Xue F, Zhang W (2018). Exon 3 mutations of ctnnb1 drive tumorigenesis: a review. Oncotarget.

[15] Wong NA, Pignatelli M (2002). Beta-Catenin--A Linchpin In Colorectal Carcinogenesis. Am J Pathol.

[16] Machin P, Catasus L, Pons C, Muñoz J, Matias-Guiu X, Prat J (2002). Ctnnb1 mutations and beta-catenin expression in endometrial carcinomas. Hum Pathol.

[17] Provost E, Mccabe A, Stern J, Lizardi I, D’aquila TG, Rimm Dl. Functional Correlates Of Mutation Of The Asp32 And Gly34 Residues Of Beta-Catenin. Oncogene. 2005;24(16):2667–76. 10.1038/sj.onc.1208346 15829978

[18] Provost E, Yamamoto Y, Lizardi I (2003). Functional correlates of mutations in beta-catenin exon 3 phosphorylation sites. J Biol Chem.

