# Supplementary material for: CTNNB1 p.D32A (c.95A > C) somatic mutation in stage I grade 1 endometrioid endometrial carcinoma with lung metastasis: a case report
Source: BMC Med Genomics. 2023 Jun 16;16:137. doi: 10.1186/s12920-023-01570-3 (PMC10273753; doi:10.1186/s12920-023-01570-3)
Supplement: Supplementary file 1 — Additional file 1: Supplementary Table 1. All genes sequenced. [file 12920_2023_1570_MOESM1_ESM.docx]

Supplementary Table 1. All genes sequenced.

| *ABCB1* | *CCND2* | *DSCAM* | *FGF3* | *HNF1A* | *MAP3K13* | *NHEJ1* | *POU5F1* | *RYBP* | *TAP2* |
| --- | --- | --- | --- | --- | --- | --- | --- | --- | --- |
| *ABCG2* | *CCND3* | *DUSP4* | *FGF4* | *HOXB13* | *MAP3K14* | *NKX2-1* | *PPARG* | *RYR2* | *TBL1XR1* |
| *ABL1* | *CCNE1* | *DUT* | *FGF6* | *HRAS* | *MAP4K3* | *NKX3-1* | *PPM1D* | *RYR3* | *TBX3* |
| *ABRAXAS1* | *CD74* | *DYNC2H1* | *FGF10* | *HSD3B1* | *MAPK1* | *NLRP1* | *PPP2R1A* | *SCG5* | *TCF3* |
| *ACSL3* | *CD79B* | *E2F3* | *FGF12* | *HSD17B4* | *MAPK3* | *NOTCH1* | *PPP2R2A* | *SDC4* | *TCF4* |
| *ACVR1* | *CD274* | *EDC4* | *FGF14* | *HSP90AA1* | *MAPKAP1* | *NOTCH2* | *PPP4R2* | *SDHA* | *TCF7L2* |
| *ACVR2A* | *CD276* | *EGFR* | *FGF19* | *HSPA4* | *MAX* | *NOTCH3* | *PPP6C* | *SDHAF2* | *TEK* |
| *ACYP2* | *CDC27* | *EIF1AX* | *FGFR1* | *ICOSLG* | *MB21D2* | *NOTCH4* | *PRDM1* | *SDHB* | *TERT* |
| *ADGRA2* | *CDC42* | *EIF4A2* | *FGFR2* | *ID3* | *MC1R* | *NPM1* | *PRDM14* | *SDHC* | *TET1* |
| *AFF4* | *CDC73* | *ELAC2* | *FGFR3* | *IDH1* | *MCL1* | *NQO1* | *PREX2* | *SDHD* | *TET2* |
| *AJUBA* | *CDH1* | *ELF3* | *FGFR4* | *IDH2* | *MDC1* | *NR4A3* | *PRKAR1A* | *SEMA3C* | *TFE3* |
| *AKT1* | *CDH9* | *ELOC* | *FH* | *IFNGR1* | *MDH2* | *NRAS* | *PRKCI* | *SESN1* | *TGFBR1* |
| *AKT2* | *CDK4* | *EME1* | *FLCN* | *IGF1* | *MDM2* | *NSD1* | *PRKD1* | *SESN2* | *TGFBR2* |
| *AKT3* | *CDK6* | *EME2* | *FLI1* | *IGF1R* | *MDM4* | *NSD2* | *PRKDC* | *SESN3* | *TIPARP* |
| *ALK* | *CDK8* | *EML4* | *FLNA* | *IGF2* | *MECOM* | *NSD3* | *PRKN* | *SETD2* | *TMEM127* |
| *AMER1* | *CDK12* | *EMSY* | *FLT1* | *IGF2R* | *MED12* | *NT5C2* | *PRPF40B* | *SF3B1* | *TMPRSS2* |
| *APC* | *CDKN1A* | *EP300* | *FLT3* | *IKBKE* | *MEF2B* | *NTHL1* | *PRSS1* | *SGK1* | *TNFAIP3* |
| *APOB* | *CDKN1B* | *EPCAM* | *FLT4* | *IKZF1* | *MEN1* | *NTRK1* | *PTCH1* | *SH2B3* | *TNFRSF14* |
| *AR* | *CDKN1C* | *EPHA2* | *FOXA1* | *IL7R* | *MERTK* | *NTRK2* | *PTCH2* | *SH2D1A* | *TNFSF11* |
| *ARAF* | *CDKN2A* | *EPHA3* | *FOXL2* | *IL10* | *MET* | *NTRK3* | *PTEN* | *SHOC2* | *TOP1* |
| *ARID1A* | *CDKN2B* | *EPHA4* | *FOXO1* | *INHA* | *MGA* | *NUDT18* | *PTGIS* | *SHPRH* | *TOP3A* |
| *ARID1B* | *CDKN2C* | *EPHB1* | *FOXP1* | *INHBA* | *MGMT* | *NUF2* | *PTP4A1* | *SHQ1* | *TOPBP1* |
| *ARID2* | *CDRT4* | *EPPK1* | *FRAS1* | *INPP4A* | *MITF* | *NUTM1* | *PTPN11* | *SIPA1* | *TP53* |
| *ASXL1* | *CDX2* | *ERBB2* | *FUBP1* | *INPP4B* | *MKNK1* | *NYAP2* | *PTPRD* | *SLC7A8* | *TP53BP1* |
| *ATAD2* | *CEBPA* | *ERBB3* | *FYN* | *INSR* | *MLH1* | *PAK1* | *PTPRO* | *SLC28A3* | *TP63* |
| *ATF1* | *CETN2* | *ERBB4* | *G6PC* | *IRF2* | *MLH3* | *PAK5* | *PTPRS* | *SLC34A2* | *TPM3* |
| *ATM* | *CFTR* | *ERCC1* | *GAB2* | *IRF4* | *MMS19* | *PALB2* | *PTPRT* | *SLC45A3* | *TRAF2* |
| *ATR* | *CHD1* | *ERCC2* | *GABRA6* | *IRS2* | *MPL* | *PARP1* | *QKI* | *SLCO1B1* | *TRAF7* |
| *ATRX* | *CHEK1* | *ERCC3* | *GALNT12* | *JAK1* | *MRE11* | *PARP2* | *RAB35* | *SLX1A* | *TRRAP* |
| *AURKA* | *CHEK2* | *ERCC4* | *GATA1* | *JAK2* | *MS4A1* | *PARP3* | *RAC1* | *SLX4* | *TSC1* |
| *AURKB* | *CIC* | *ERCC5* | *GATA2* | *JAK3* | *MSH2* | *PARP4* | *RAC2* | *SMAD2* | *TSC2* |
| *AXIN1* | *CLK2* | *ERCC6* | *GATA3* | *JMJD1C* | *MSH3* | *PAX5* | *RAD21* | *SMAD3* | *TSHR* |
| *AXIN2* | *COL11A1* | *ERF* | *GATA4* | *JUN* | *MSH4* | *PAX8* | *RAD50* | *SMAD4* | *TUBB3* |
| *AXL* | *COL22A1* | *ERG* | *GATA6* | *KDM5C* | *MSH5* | *PBRM1* | *RAD51* | *SMARCA1* | *TYMS* |
| *B2M* | *COP1* | *ERRFI1* | *GEN1* | *KDM6A* | *MSH6* | *PBX1* | *RAD51B* | *SMARCA4* | *U2AF1* |
| *BABAM2* | *CREB1* | *ESR1* | *GGH* | *KDR* | *MSI1* | *PCDH9* | *RAD51C* | *SMARCB1* | *UGT1A1* |
| *BACH1* | *CREBBP* | *ETV1* | *GID4* | *KEAP1* | *MSI2* | *PDCD1* | *RAD51D* | *SMARCD1* | *UMPS* |
| *BAP1* | *CRKL* | *ETV4* | *GLI1* | *KIAA1549* | *MST1* | *PDCD1LG2* | *RAD52* | *SMO* | *UNC5D* |
| *BARD1* | *CSDE1* | *ETV5* | *GNA11* | *KIF1B* | *MST1R* | *PDGFRA* | *RAD54B* | *SMYD3* | *UPF1* |
| *BCL2* | *CSF1R* | *ETV6* | *GNAQ* | *KIF5B* | *MTAP* | *PDGFRB* | *RAD54L* | *SNCAIP* | *USP6* |
| *BCL2A1* | *CSMD3* | *EWSR1* | *GNAS* | *KIT* | *MTDH* | *PDK1* | *RAF1* | *SOCS1* | *VEGFA* |
| *BCL2L1* | *CTCF* | *EXO1* | *GPS2* | *KLF6* | *MTHFR* | *PGR* | *RARA* | *SOD2* | *VHL* |
| *BCL6* | *CTLA4* | *EXOC2* | *GRB7* | *KLHL6* | *MTOR* | *PHF6* | *RASA1* | *SOS1* | *VTCN1* |
| *BCOR* | *CTNNA1* | *EXT1* | *GREM1* | *KLLN* | *MTRR* | *PHOX2B* | *RB1* | *SOX2* | *WEE1* |
| *BCR* | *CTNNB1* | *EXT2* | *GRIN2A* | *KMT2A* | *MUC6* | *PIK3CA* | *RBBP8* | *SOX4* | *WRN* |
| *BIRC2* | *CTNND2* | *EZH1* | *GRM3* | *KMT2B* | *MUC16* | *PIK3CB* | *RBM10* | *SOX9* | *WT1* |
| *BIRC3* | *CUL3* | *EZH2* | *GSK3B* | *KMT2C* | *MUS81* | *PIK3CG* | *RECQL* | *SOX10* | *WWTR1* |
| *BLM* | *CUL4A* | *EZR* | *GSTP1* | *KMT2D* | *MUTYH* | *PIK3R1* | *RECQL4* | *SOX17* | *XIAP* |
| *BMPR1A* | *CUL4B* | *FAM135B* | *H1-2* | *KMT5A* | *MYB* | *PIK3R2* | *REEP5* | *SPEN* | *XPA* |
| *BRAF* | *CXCR4* | *FAN1* | *H2AX* | *KNSTRN* | *MYC* | *PIK3R3* | *REL* | *SPINK1* | *XPC* |
| *BRCA1* | *CYLD* | *FANCA* | *H2BC5* | *KRAS* | *MYCL* | *PIM1* | *RET* | *SPOP* | *XPO1* |
| *BRCA2* | *CYP2C8* | *FANCB* | *H3-3A* | *LAMA2* | *MYCN* | *PLAG1* | *RFC4* | *SPOPL* | *XRCC1* |
| *BRCC3* | *CYP2D6* | *FANCC* | *H3-3B* | *LATS1* | *MYD88* | *PLCG2* | *RHEB* | *SPRED1* | *XRCC2* |
| *BRD4* | *CYP11B1* | *FANCD2* | *H3-4* | *LATS2* | *MYOD1* | *PLK1* | *RHOA* | *SRC* | *XRCC3* |
| *BRF1* | *CYP17A1* | *FANCE* | *H3C1* | *LHCGR* | *MYSM1* | *PLK2* | *RICTOR* | *SRSF2* | *YAP1* |
| *BRIP1* | *CYP19A1* | *FANCF* | *H3C2* | *LIFR* | *NABP2* | *PLXNA1* | *RIT1* | *STAG1* | *YES1* |
| *BTK* | *DAXX* | *FANCG* | *H3C3* | *LIG4* | *NBN* | *PMAIP1* | *RNF43* | *STAG2* | *YWHAZ* |
| *C8orf34* | *DCUN1D1* | *FANCI* | *H3C4* | *LRP1B* | *NCOA2* | *PMS1* | *ROS1* | *STAT3* | *ZBTB16* |
| *CARD11* | *DDB2* | *FANCL* | *H3C6* | *LRRK1* | *NCOA3* | *PMS2* | *RPS6KA3* | *STAT5A* | *ZFHX3* |
| *CARM1* | *DDR1* | *FANCM* | *H3C7* | *LRRK2* | *NCOA4* | *PNPLA3* | *RPS6KA4* | *STAT5B* | *ZFHX4* |
| *CASP8* | *DDR2* | *FAT1* | *H3C8* | *LTK* | *NCOR1* | *PNRC1* | *RPS6KB2* | *STK11* | *ZMYM3* |
| *CASR* | *DICER1* | *FAT2* | *H3C10* | *LYN* | *NCOR2* | *POLD1* | *RRAGC* | *STK19* | *ZNF2* |
| *CBL* | *DIS3* | *FAT3* | *H3C11* | *LZTR1* | *NEGR1* | *POLE* | *RRAS* | *STK40* | *ZNF217* |
| *CBLB* | *DMC1* | *FAT4* | *H3C13* | *MALT1* | *NEIL2* | *POLG* | *RRAS2* | *SUFU* | *ZNF703* |
| *CBR3* | *DNMT3A* | *FBXW7* | *H3C14* | *MAP2K1* | *NF1* | *POLH* | *RSPO2* | *SUZ12* | *ZNF770* |
| *CBX4* | *DNTT* | *FCGR2B* | *HDAC1* | *MAP2K2* | *NF2* | *POLM* | *RTEL1* | *SYK* | *ZNRF3* |
| *CCDC6* | *DOCK2* | *FCGR3A* | *HGF* | *MAP2K4* | *NFE2L2* | *POLN* | *RUFY4* | *TAF1L* | *ZRSR2* |
| *CCNA2* | *DOT1L* | *FGD4* | *HLA-A* | *MAP3K1* | *NFKB1* | *POLQ* | *RUNX1* | *TAF15* |  |
| *CCND1* | *DPYD* | *FGF2* | *HLA-B* | *MAP3K4* | *NFKBIA* | *POT1* | *RXRA* | *TAP1* |  |
