# Supplementary material for: CTNNB1 p.D32A (c.95A > C) somatic mutation in stage I grade 1 endometrioid endometrial carcinoma with lung metastasis: a case report
Source: BMC Med Genomics. 2023 Jun 16;16:137. doi: 10.1186/s12920-023-01570-3 (PMC10273753; doi:10.1186/s12920-023-01570-3)
Supplement: Supplementary file 2 — Additional file 2: Supplementary Table 2. list of mutations in primary and lung metastatic tumors. [file 12920_2023_1570_MOESM2_ESM.doc]

Supplementary Table 2. Mutation list of primary and lung metastasis tumors.

| **Primary Tumor** | | **Lung Metastasis** | |
| --- | --- | --- | --- |
|  | **Frequency** |  | **Frequency** |
| | ***PTEN*** | p.P248Lfs*8 (c.743delC) |  | | --- | --- | --- | | 14.77% | | ***PTEN*** | p.P248Lfs*8 (c.743delC) |  | | --- | --- | --- | | 8.67% |
| | ***CTNNB1*** | p.D32A (c.95A>C) | | --- | --- | | 7.85% | | ***CTNNB1*** | p.D32A (c.95A>C) |  | | --- | --- | --- | | 4.44% |
| | ***NF1*** | p.R440* (c.1318C>T) | | --- | --- | | 1.21% | | ***BCOR*** | p.N1425S (c.4274A>G) |  | | --- | --- | --- | | 5.63% |
| | ***BACH1*** | p.R391* (c.1171C>T) |  | | --- | --- | --- | | 1.45% | | ***CBL*** | p.S439N (c.1316G>A) |  | | --- | --- | --- | | 5.23% |
| | ***KDM6A*** | p.R172* (c.514C>T) |  | | --- | --- | --- | | 1.19% | | ***PIK3CA*** | p.L113_I117del (c.336_350del) |  | | --- | --- | --- | | 3.1% |
| | ***KMT2D*** | p.Q1029* (c.3085C>T) |  | | --- | --- | --- | | 1.15% |  |  |
| | ***RAD54B*** | p.R560* (c.1678C>T) |  | | --- | --- | --- | | 1.08% |  |  |
| | ***ZNF217*** | p.R752* (c.2254C>T) | | --- | --- | | 1.07% |  |  |
| | ***BCOR*** | p.N1425S (c.4274A>G) | | --- | --- | | 9.11% |  |  |
| | ***KMT2B*** | p.R218W (c.652C>T) |  | | --- | --- | --- | | 6.12% |  |  |
| | ***CBL*** | p.S439N (c.1316G>A) |  | | --- | --- | --- | | 6.05% |  |  |
| | ***PIK3CA*** | p.L113_I117del (c.336_350del) |  | | --- | --- | --- | | 3.22% |  |  |
| | ***LAMA2*** | p.S157C (c.470C>G) |  | | --- | --- | --- | | 2.42% |  |  |
| | ***FBXW7*** | p.R441Q (c.1322G>A) | | --- | --- | | 2.25% |  |  |
| | ***GEN1*** | c.1408+1G>A | | --- | --- | | 2.18% |  |  |
| | ***MYB*** | p.R454H (c.1361G>A) | | --- | --- | | 1.87% |  |  |
| | ***BACH1*** | p.E281K (c.841G>A) | | --- | --- | | 1.85% |  |  |
| | ***MSH2*** | p.S860L (c.2579C>T) | | --- | --- | | 1.69% |  |  |
| | ***CDK12*** | p.R202Q (c.605G>A) | | --- | --- | | 1.68% |  |  |
| | ***FAT3*** | p.E2774K (c.8320G>A) | | --- | --- | | 1.63% |  |  |
| | ***ERCC5*** | p.E426K (c.1276G>A) | | --- | --- | | 1.54% |  |  |
| | ***ZFHX4*** | p.E3213K (c.9637G>A) | | --- | --- | | 1.51% |  |  |
| | ***KIAA1549*** | p.A220V (c.659C>T) | | --- | --- | | 1.48% |  |  |
| | ***ARID2*** | p.R1504Q (c.4511G>A) | | --- | --- | | 1.38% |  |  |
| | ***POLE*** | p.R1932C (c.5794C>T) | | --- | --- | | 1.37% |  |  |
| | ***MUC16*** | p.R6274H (c.18821G>A) | | --- | --- | | 1.32% |  |  |
| | ***TCF7L2*** | p.E26K (c.76G>A) | | --- | --- | | 1.29% |  |  |
| | ***NCOR1*** | p.P919L (c.2756C>T) | | --- | --- | | 1.23% |  |  |
| | ***CDK12*** | p.G720E (c.2159G>A) | | --- | --- | | 1.23% |  |  |
| | ***FAT1*** | p.V41I (c.121G>A) | | --- | --- | | 1.19% |  |  |
| | ***BRCA2*** | p.E58K (c.172G>A) | | --- | --- | | 1.17% |  |  |
| | ***EXT2*** | p.V115M (c.343G>A) | | --- | --- | | 1.16% |  |  |
| | ***CDK12*** | p.R1356Q (c.4067G>A) | | --- | --- | | 1.16% |  |  |
| | ***SPEN*** | p.P2432S (c.7294C>T) | | --- | --- | | 1.15% |  |  |
| | ***KMT2A*** | p.R3545W (c.10633C>T) | | --- | --- | | 1.12% |  |  |
| | ***PTPRT*** | p.R470Q | | --- | --- | | 1.12% |  |  |
| | ***NOTCH1*** | p.E756K (c.2266G>A) | | --- | --- | | 1.11% |  |  |
| | ***U2AF1*** | p.R118C (c.352C>T) | | --- | --- | | 1.11% |  |  |
| | ***FAT4*** | p.T3163M (c.9488C>T) | | --- | --- | | 1.1% |  |  |
| | ***FLCN*** | p.R414W (c.1240C>T) | | --- | --- | | 1.09% |  |  |
| | ***SUFU*** | p.G378R (c.1132G>A) | | --- | --- | | 1.08% |  |  |
| | ***RYR2*** | p.V452I (c.1354G>A) | | --- | --- | | 1.07% |  |  |
| | ***CTNNA1*** | p.D661N (c.1981G>A) | | --- | --- | | 1.06% |  |  |
| | ***NSD3*** | p.R357Q (c.1070G>A) | | --- | --- | | 1.06% |  |  |
